# Research on the quality changes of grass carp during brine salting

**DOI:** 10.1002/fsn3.1599

**Published:** 2020-05-13

**Authors:** Wenxian Yang, Wenzheng Shi, Yinghong Qu, Zhihe Wang, Siyuan Shen, Ludan Tu, Haiyuan Huang, Han Wu

**Affiliations:** ^1^ College of Food Science and Technology Shanghai Ocean University Shanghai China; ^2^ National R&D Branch Center for Freshwater Aquatic Products Processing Technology (Shanghai) Shanghai China

**Keywords:** ATP‐related compounds, brine salting, equivalent umami concentration, free amino acids, taste activity value

## Abstract

The research on the quality changes of grass carp during brine salting with 6%, 8%, and 10% salt additions was evaluated by chemical and physical indicators, as well as a sensory assessment and microbiological analysis. The NaCl content was proportional to salt addition and salting time. The increase of salt addition could lead to the increase of hardness and chewiness in which change trends were contrary to the pH within 24 hr. All K values were less than 10% during brine salting. The effect of 8% salt additions on free amino acids was relatively smaller. Higher levels of salt additions could inhibit bacterial growth. Combined with sensory assessment, equivalent umami concentration (EUC), and taste activity value (TAV) to analysis comprehensively, it was suggested that grass carp meat should be eaten at 4–8 hr of brine salting with 8% salt additions or processed for the next step, in which the grass carp meat had a better taste and quality.

## Graphical abstract

The quality changes of grass carp meat were studied over a period of 48 hr of brine salting with different salt additions (6%, 8%, and 10% (w/w)) by evaluating the K values, levels of free amino acids, EUC and TAV, as well as a sensory assessment, microbiological analysis, and electronic tongue evaluation. In addition to establish a firm theoretical foundation for quality control of grass carp during salting, these results would provide useful information for improving the nutritional value of aquatic products.

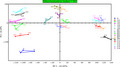

## 1 INTRODUCTION

Grass carp (*Ctenopharyngodon idellus*) is an important freshwater fish in the world because of its fast growth rate, easy cultivation, high nutritional value, and relatively low price. Total global production of grass carp was about 6.06 million tons in 2016 (FAO, 2018), the highest for any aquaculture species. As production of aquatic products increases, the quality requirements of consumers are also increasing. Meanwhile, reducing the quality loss after harvesting becomes ever more important. Generally, grass carp has been used mainly for fresh consumption. Due to the current high volume of production, it could be used to develop other process products.

Salting is one of the oldest ways to preserve or prepared process fish. It is based on the penetration of salt into fish muscles. Nowadays, salting is considered to give the final product‐specific sensory characteristics (Boudhrioua, Djendoubi, Bellagha, & Kechaou, 2009). When the salt concentration is less than 20%, it can improve the color and appearance of salted fish products (Thorarinsdottir, Arason, Bogason, & Kristbergsson, 2004). Dry salting and brine salting are the two main salting methods. Compared with dry salting, brine salting has several advantages, including shorter processing time due to higher salt uptake, and higher weight yield due to better control of salt uptake and water loss in muscle (Andrés, Rodríguez‐Barona, Barat, & Pedro, 2004). Salting can extend the shelf life of fish by reducing the activity of water and the chance of microbial attack and enhancing functional properties of fish protein (Yanar, Celik, & Akamca, 2006). Water loss and salt uptake are usually generated during salting which could induce the quality changes of the salted fish because of the changes of lipid, protein, and other components in fish muscle (Barat, Rodríguez‐Barona, Andrés, & Fito, 2003). The concentration of saline affects the rate at which salt spreads to muscles and the amount of protein extracted. Proteins may denature at high salt concentration, resulting in muscle contraction and dehydration (Thorarinsdottir, Arason, Geirsdottir, Bogason, & Kristbergsson, 2002). In addition, high uptakes of salt can lead to some chronic diseases, like hypertension and cardiovascular diseases (Gallart‐Jornet, Rustad, Barat, Fito, & Escriche, 2007). Light salting fish has been used to increase the flavor of food, to reduce drip loss, and to counteract the negative effects of freezing (Gudjónsdóttir et al., 2011). The uptake and distribution of salt largely depend on the method used, salt concentration, and salting time (Birkeland, Akse, Joensen, Tobiassen, & Skara, 2007).

There have been many studies on physicochemical properties and quality changes of aquatic products during salting. Chaijan (2011) investigated the effect of wet and dry salting on the physicochemical changes of tilapia muscle and found that dry salting resulted in a higher oxidation of tilapia muscle lipid throughout the salting period. Fan, Luo, and Yin (2014) believed that salting treatments could reduce chemical changes reflected in HxR, Hx, pH, and total volatile base nitrogen, decrease cooking loss, and increase overall sensory quality of black carp fillets stored at 4°C. Qin et al. (2017) studied the influence of lightly salting (dry salting) and sugaring on the quality and water distribution of grass carp during super‐chilled storage, and the results showed that salting treatments predominately accelerated IMP formation, inhibited bacterial growth, retarded cooking loss, and improved the overall sensory quality. There also have been many reports focused on the grass carp, including volatile flavor substances of grass carp and studies of evaluating the quality changes of grass carp majorly involving the different season (Chen, Fang, Shi, & Chen, 2017) and storage process (Wang, Zhu, Zhang, Wang, & Shi, 2018) and so on. However, the report of the effect of brine salting on the quality changes in grass carp muscle was scarce. Therefore, the research mainly focused on the quality changes of grass carp meat during brine salting with different salt additions will be of certain significance.

The products of salted grass carp can be steamed or fried, which are traditional Chinese dishes and in which fish fillets are salted in brine first generally. Salt concentration and salting time have great influences on the quality change of fish and the flavor of the final product during the brine salting process. Therefore, the main objective of this work was to study the quality changes of grass carp meat during brine salting with different salt additions (6%, 8%, and 10% (w/w)). This was achieved by evaluating the meat moisture content, sodium chloride content, texture properties, color changes and pH, ATP‐related compounds, K value, and levels of free amino acids, as well as a sensory assessment, microbiological analysis, and electronic tongue evaluation. In addition, the indicators of equivalent umami concentration and taste activity value were analyzed. It was desired to establish a firm theoretical foundation for the quality control of grass carp during salting. These will also promote the study of the mechanism of flavor changes of aquatic products in the process of salting.

## 2 MATERIALS AND METHODS

### 2.1 Sample preparation and Salting

Twenty fresh grass carp (weight of 2.3 ± 0.3 kg, length of 58.5 ± 3.2 cm, and width of 9.2 ± 0.7 cm) were from captured an aquaculture farm in Shanghai, China, in January 2019 and then transported alive to the laboratory within half an hour. They were stunned by a physical blow to the head, gutted, beheaded, scaled, washed, drained, and cut into skin‐on fillets with an average weight of 3.5 ± 3 g (about 3.5 × 3.5 × 2.5 cm).

The fillets were collected randomly and salted by immersing them in saline with three different salt concentrations (6%, 8%, and 10%) at a fish‐to‐brine ratio of 1:1.5. Noniodized food grade salt (Shanghai, China) and distilled water were used to make the saline. The salting process was performed at 4°C for 48 hr. Samples from each sampling point (0, 4, 8, 16, 24, 36, and 48 hr) were brined in one closed plastic container, and ten fillets were chosen randomly at each sampling point for analyses. The brined fillets were drained for 2–3 min on grid before determining.

### 2.2 Determination of moisture content and NaCl content

Moisture content was determined using oven‐dry method at 105 ± 1°C until constant weight (ISO‐6496, 1999). Sodium chloride (NaCl) content was determined by the method of Volhard (ISO‐, 1841‐1, 1996).

### 2.3 Determination of muscle pH

The determination of muscle pH was determined using the method described by Fan et al. (2014) and modified slightly. A sample (2.00 g) of salting fillets was dispersed in 18 ml of distilled water and centrifuged at 10,000 g for 10 min, and then filtered. The pH value of the filtrate was determined by a digital pH meter (Mettler Toledo FE20/EL20) and performed in triplicate.

### 2.4 Microbiological analysis

Samples were weighed 5.00 g aseptically and homogenized (Shanghai Huxi Industrial Co. Ltd., Shanghai, China) with 45 ml of sterilized 0.85% (w/v) physiological saline (Sinopharm Chemical Reagent, Shanghai, China) for 2 min. A series of 1:10 (v/v) dilutions were made by serially diluted with sterile saline for bacteriological analyses. One mL of serial dilution was spread onto plate count agar, and the indicator of total viable counts (TVC) was measured after incubating at 30°C for 72 hr. All counts were performed in duplicate and expressed as log10 CFU/g. The total viable count was calculated according to the Chinese standard method of GB‐4789.2 (2016).

### 2.5 Sensory assessment

The fillets of salted grass carp meat were immersed in pure water for 15 min and steamed for 8 min. When the steamed samples were cooled to about 40°C, sensory assessment was conducted from five aspects (salty, odor, morphology, mouthfeel, and color) by ten trained sensory assessment personnel (4 males and 6 females, average age 25 years old) using standard sensory assessment (SA) methods with slight modifications (Table 1; Gao, Zhu, Wu, Li, & Zhang, 2013; Gu et al., 2018). According to the National Standards No. 16,291.1–2012 of the People's Republic of China, the ten sensory evaluators were selected to conduct sensory experiments in the sensory assessment laboratory of Shanghai Ocean University (established jointly with Pepsi Food Co., Ltd. [China] in 2001). The total score of 100 points was deemed best and below 60 points was unacceptable.

**TABLE 1:** Standards of sensory assessment applied for the salted grass carp

Quality parameters	Description points				
	1–4	5–8	9–12	13–16	17–20
Salty	Unacceptable	Very salty	Slight salty	Moderate salty	Excellent
Odor	Unacceptable	off‐odor	Slightly off‐odor	Desirable	Extremely desirable
Appearance	Dull	Slight dull	Slight bright	Bright	Very bright
Morphology	Very loose	Loose	Partial loose	Tight	Very tight
Texture	Very soft or stiff	Soft	Slight elastic	Elastic	Firm

### 2.6 Texture measurements

The transverse slices of salted fish fillet were taken and were cut into 2 × 2 × 1 cm pieces. Textural properties were measured by a TA‐XT Plus (Stable Micro System) equipped with a P/6 cylindrical probe. The test was performed by twice compression (50%) with an interval of 5 s at a constant speed of 1.0 mm/s carried by probe (Tang, Xie, Xu, Zhang, & Gao, 2015). The trigger value was 5 g, and the data collection rate was 200 PPS. The values of hardness, springiness, cohesiveness, chewiness, and resilience were calculated as described by Bourne (1978) and measured with eight replications.

### 2.7 Color measurements

The colorimetric values of the fish sample were obtained by using an automatic chromaticity instrument (CR‐400, Konica Minolta, Japan), which was calibrated with standard white board before sample determination. The sample was cut into slices of uniform size and 1 cm thickness, and each group of samples was measured 6 times. The results were expressed in L* (lightness), a* (redness/ greenness), and b* (yellowness/blueness) (Gerdes & Valdez, 1991).

### 2.8 ATP‐related compounds and K value

The extraction method of ATP‐related compounds was improved slightly according to the previous methods (Wang et al., 2018; Yokoyama, Sakaguchi, Kawai, & Kanamori, 1992). The sample (5.00 g) was homogenized with 10 ml of cold perchloric acid (10% (v/v)) by using FM‐200 homogenizer (Shanghai Fokker Equipment Co. Ltd.) for 30 s and centrifuged by using H2050R high‐speed freezing centrifuge (Changsha Xiangyi Co. Ltd.) at 10,000 *g* for 15 min at 4°C. The precipitate was washed with 5 ml cold perchloric acid (5% (v/v)) and centrifuged under the same conditions, which was repeated twice. The pH of combined supernatant was adjusted to 6.5 with potassium hydroxide solutions (1 and 10 M) and stand for 30 min. The supernatant was diluted to 50 ml with ultrapure water and filtered with a 0.45‐μm membrane. All above operations were carried out below 4°C.

ATP‐related compounds were analyzed by high‐performance liquid chromatography (HPLC) (Waters Co.) equipped with COSMOSIL 5C18‐PAQ column (4.6 ID × 250 mm) (GL Sciences, Inc., Tokyo, Japan) and SPD‐10A (V) detector. The 0.05 M phosphate buffer (pH 6.5) and methanol were used for mobile phase to elute with a flow rate of 1 ml/min for 20 min. The injection volume was 10 μl, and detection wavelength was 254 nm. The calculation formula of K value is as follows (Saito, Arai, & Matsuyoshi, 1959): K value (%) = [(HxR + Hx)/ (ATP + ADP +AMP + IMP +HxR + Hx)] × 100.

### 2.9 Free amino acids

Free amino acids (FAA) were extracted according to the procedure described by Yu et al. (2018) and slightly modified. The sample (2.00 g) was homogenized with 15 ml trichloroacetic acid solution (15% (v/v)) for 2 min and stand for 2 hr, and then centrifuged at 10,000 g for 15 min below 4°C. The supernatant (5 ml) was adjusted pH to 2.0 with sodium hydroxide solution (3 M), diluted to 10 ml with ultrapure water, and filtered with a 0.22‐μm membrane. All above operations were carried out below 4°C.

Free amino acids were determined and analyzed by automatic amino acid analyzer (L‐8800, Hitachi, Japan). The mobile phase was a mixed buffer solution (pH 3.2, 3.3, 4.0, and 4.9) of sodium citrate and citric acid and a ninhydrin buffer (4% (w/v)). Flow rates were 0.4 ml/min and 0.35 ml/min, respectively.

### 2.10 Taste active value and equivalent umami concentration

Taste active value (TAV) is the ratio of the concentration of flavor substance in sample to the corresponding threshold, which is widely used in various food flavor studies (Scharbert & Hofmann, 2005). The taste active substance has a significant contribution to the overall taste of the sample when TAV is greater than 1, and its value is proportional to the contribution. On the contrary, the taste active substance has little contribution to the overall taste of the sample when TAV is less than 1 (Shi, Fang, Wu, Pan, & Hou, 2017).

Equivalent umami concentration (EUC) means that the umami intensity produced by the synergistic effect of umami amino acids (Glu and Asp) and flavor nucleosides (IMP, AMP, etc.) equivalent to the concentration of a single monosodium glutamate (MSG) (Shi et al., 2017). The value of EUC was calculated following the formula (Yamaguchi, Yoshikawa, Ikeda, & Ninomiya, 1971): EUC = ∑a_i_b_i_+1,218(∑a_i_b_i_) × (∑a_j_b_j_).

In the formula, EUC is equivalent umami concentration (gMSG/100 g), ai is the concentration of umami amino acid (Asp or Glu) (g/100 g), bi is the umami coefficient of umami amino acid relative to MSG (Glu: 1, Asp: 0.077), aj is the concentration of flavor nucleotide (5’‐IMP, 5’‐AMP) (g/100 g), bj is the umami coefficient of flavor nucleotide relative to IMP (5’‐IMP: 1, 5’‐AMP: 0.18), and 1,218 is the synergy constant.

### 2.11 Electronic tongue

The principal component analysis (PCA) of electronic tongue (Alpha M.O.S., ASTREE) was generally used to analyze the taste of sample. The determination was according to the method described by Zhang, Gu, Ding, Wang, and Jiang (2015) and slightly modified. The sample (2.00 g) was homogenized with 25 ml deionized water for 2 min and stand for 30 min, and then centrifuged at 10,000 g for 10 min at 4°C. The supernatant was diluted to 100 ml with deionized water. The diluted solution (5 ml) was diluted to 80 ml with deionized water and placed it into a special sampling cup of electronic tongue for testing.

The electronic tongue system was equipped with seven sensors of UMS, GPS, SWS, SRS, STS, SPS, and BRS. UMS, SRS, and STS had specific responses to umami, sour, and salty taste, respectively. The sensors of electronic tongue were adjusted and calibrated with hydrochloric acid (0.01 M) before determination. In order to ensure the stability of the sensors, the data of each sample were collected once every second for 120 s. The sensors were rinsed with deionized water for 10 s between measurements to reach steady readings in water. Seven parallel analyses were performed on the each sample to keep the reliability of the results, and the data of the last three were used as the original data of PCA.

### 2.12 Statistical analysis

The software of Alpha soft 14.0 was used for PCA of electronic tongue. Other data were analyzed by the software of SPSS 24.0 (SPSS Inc., Chicago, IL, USA). The variance of the data was expressed as mean ± standard deviation. Duncan's method was used to express the results of multiple comparisons. The figures were drawn with the software of Origin 8.5 (OriginLab Corp, Hampton, USA).

## 3 RESULTS AND DISCUSSION

### 3.1 Changes in moisture content and NaCl content and muscle pH

**FIGURE 1:**
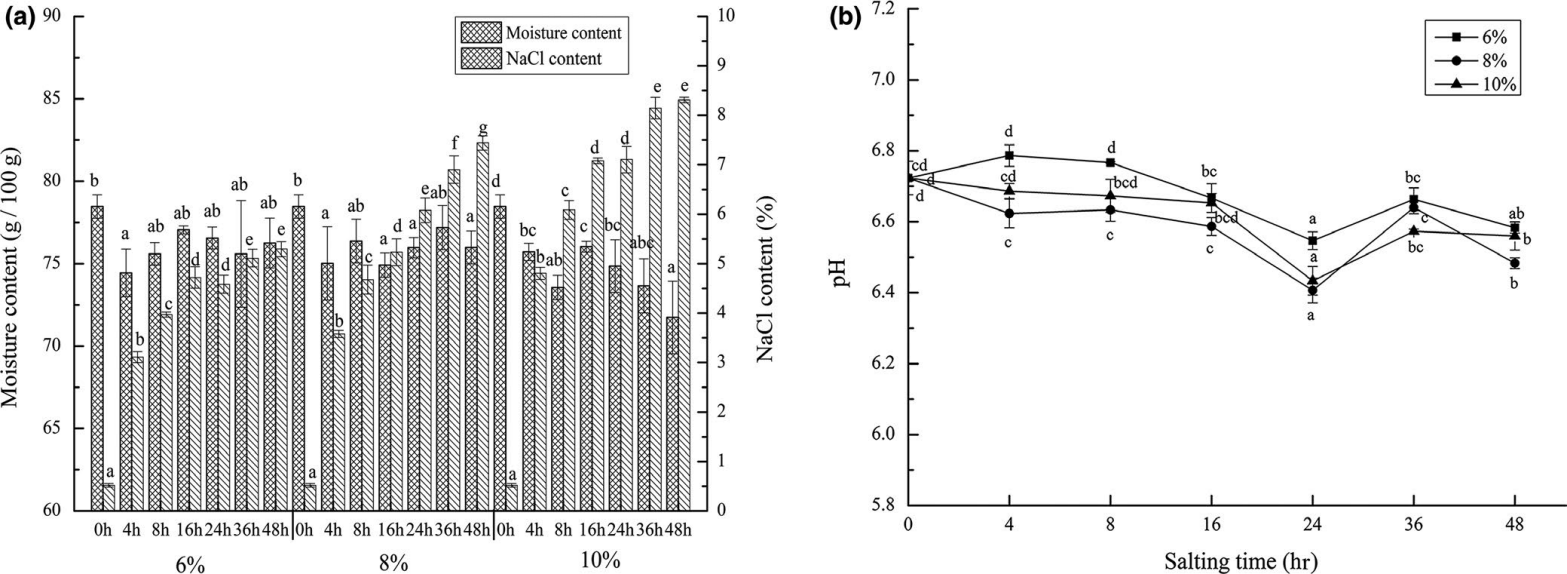
Changes in moisture and NaCl content (A) and muscle pH (B) of salting grass carp meat during brine salting with salt additions of 6%, 8%, and 10%. Data (mean ± *SD*) with different letters in the same salt addition are significantly different (*p* < .05)

Salting is a process of mass transfer between water and salt. Salt permeates into grass carp muscle through osmotic mechanism, while water diffuses from muscle through osmotic pressure (Horner, 1997). The content of salt addition is the main factor affecting the diffusion of salt and water. The higher the salt concentration is, the higher the external osmotic pressure and the higher the salt absorption rate are (Fu, 2010; Horner, 1997). From the results (Figure 1a), it can be found that the NaCl content in fish muscle increased with the extension of salting time and with the increase of salt addition in the process of salting. In addition, the NaCl content during brine salting with the salt additions of 6%, 8%, and 10% reached the maximum at 48 hr, which was 5.30%, 7.44%, and 8.31%, respectively. Chaijan (2011) immersed tilapia fillets in 25% brine for 180 min and found that the salt content of salted fish muscle gradually increased and reached the maximum value at 180 min (> 5%). Kosak and Toledo (1981) reported that salt uptake depends on a variety of factors, including fish species, weight, size, muscle thickness, muscle characteristics, physiological state, salting method, brining time, brine concentration, and fish‐to‐salt ratio. The moisture contents of grass carp meat all decreased significantly at 4 hr. From then to the end of salting, the moisture contents in the brine of 6% and 8% salt additions did not change significantly (*p* < .05). However, the moisture content was significantly changed (p＜0.05p＞0.05) during brine salting with 10% salt additions and reached the lowest value of 71.74 g/100 g at 48 hr. Jittinandana, Kenney, Slider, and Kiser (2002) believed that higher salt concentration would lead to protein precipitation and dehydration. Nketsia‐Tabiri and Sefa‐Dedeh (1995) considered that salting time was an important processing variable affecting product moisture content.

The pH changes of grass carp muscle during the salting period of 48 hr were shown in Figure 1b. The initial pH value of the fish sample was 6.72, which was close to the value of fresh ray fish (6.82) reported by Ocaño‐Higuera et al. (2011), but lower than the initial value of 6.87 when the black fish was immersed in light salt of 1.5% (Fan et al., 2014). Differences in initial pH values may be due to species, season, diet, activity levels, and other factors (Fan et al., 2014). In the process of salting with 6%, 8%, and 10% salt additions, the pH values changed in a similar trend. The pH values all decreased at different rates at first and reached the minimum values at 24 hr, which were 6.55, 6.41, and 6.43, respectively. After significant increases at 36 hr, there were downward trends. The initial decrease in pH may be due to the decomposition of glycogen, ATP, and phosphocreatine in fish muscle, while the subsequent increase may be due to endogenous or microbial enzymes (Ruiz‐capillas & Moral, 2001). It can be seen that the pH values were relatively low during brine salting with salt additions of 8% and 10%, which may be due to the growth of lactic acid bacteria. These bacteria may reduce the muscle pH by inhibiting the growth of other bacteria and buffering the basic metabolites produced (Calo‐mata et al., 2008).

### 3.2 Microbial and sensory assessment analysis

**FIGURE 2:**
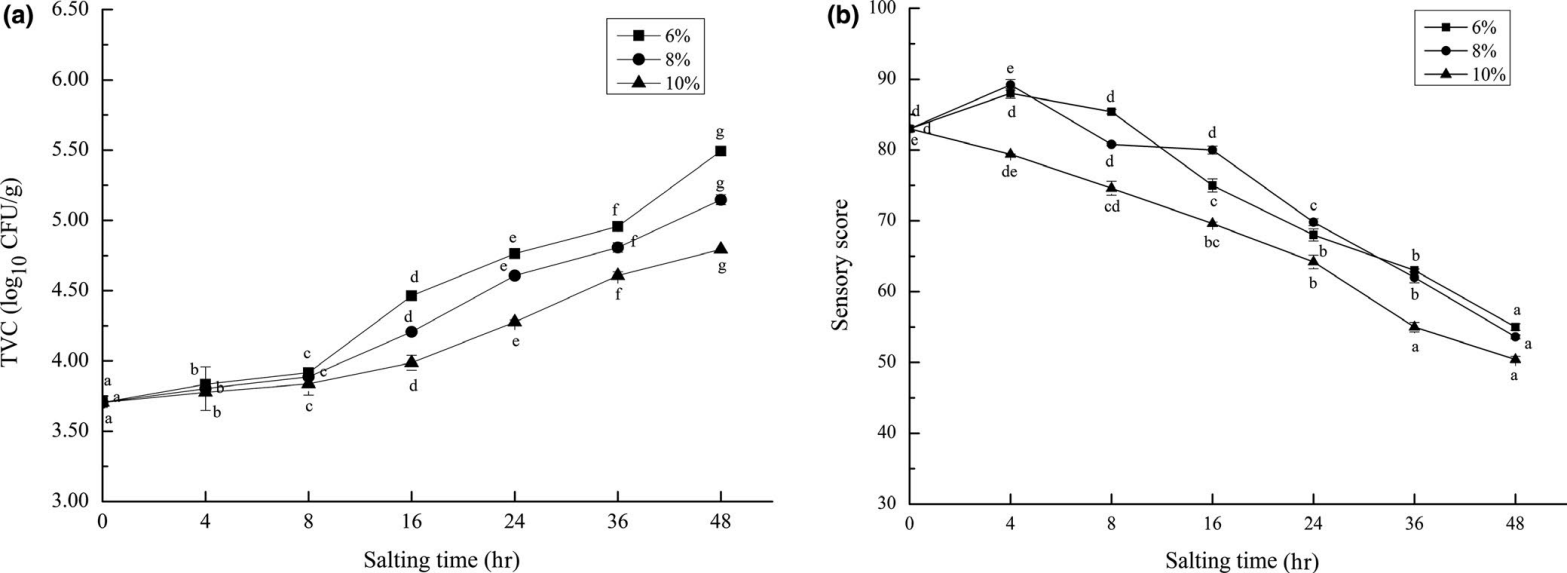
Changes in TVC (A) and sensory score (B) of salting grass carp meat during brine salting with salt additions of 6%, 8%, and 10%. Data (mean ± *SD*) with different letters in the same salt addition are significantly different (*p* < .05)

The changes in TVC of grass carp meat during the salting period of 48 hr were presented by Figure 2a. The initial TVC of grass carp was 3.70 log10 CFU/g. The black carp had an initial TVC of 3.81 log10 CFU/g when salted with 1.5% salt addition (Fan et al., 2014). Chytiri, Chouliara, Savvaidis, and Kontominas (2004) showed that the number of bacteria in freshwater fish varied between 2 and 6 log10 CFU/g with the changes of water environment and temperature. The low initial TVC in this study indicated that the quality of grass carp was better. It can be seen from Figure 2a that the TVC of grass carp meat increased with salting time and reached the maximum values at 48 hr during the brine salting with 6%, 8%, and 10% salt additions, which were 5.49 log10 CFU/g, 5.15 log10 CFU/g, and 4.79 log10 CFU/g, respectively. Ojagh, Rezaei, Razavi, and Hosseini (2010) showed that the maximum acceptable TVC value of freshwater fish was 7 log10 CFU/g, indicating that the grass carp maintained a relatively good quality during the whole salting period. In addition, it also can be found that the higher the salt addition, the slower the growth rate of TVC, indicating that salt can inhibit the growth of bacteria to a certain extent. Qin et al. (2017) also reached this conclusion when studying lightly salted grass carp meat.

It was generally believed that the four major elements of food quality are nutrition, appearance, texture, and flavor. In addition to nutritional quality, other characteristics were perceived by the senses and had an important impact on the acceptability of products (Liu, 2011). Figure 2b showed the changes in sensory scores of grass carp meat salted in brine with salt additions of 6%, 8%, and 10% for 48 hr. It can be seen that the sensory scores reached maximum at 4 hr when salted in brine with 6% and 8% salt additions, at 88.00 and 89.20, respectively, and then gradually decreased at different rates. The salting treatment reduced the appearance of grass carp fillets, but improved the taste to some extent. Fan et al. (2014) had a similar conclusion when salted black carp. However, the sensory score of grass carp meat salted with 10% salt additions decreased with salting time, and in the whole salting process, was lower than those of 6% and 8%. This indicated that the salted grass carp meat with salt additions of 6% and 8% were more acceptable due to their better taste than those of 10%. In addition, it can be seen from Figure 2b that the sensory scores in 6% and 8% salt additions decreased slowly at 4–16 hr. Combined with the microbial analysis, it may be because the higher osmotic pressure inhibited the enzyme activity and microbial growth in the muscle of grass carp. At 48 hr of salting, the sensory scores were all lower than 60, which were unacceptable to the sensory evaluators. In terms of sensory score, the qualities of grass carp meat salted with 6% and 8% salt additions were better than 10% and within 16 hr comparatively good. In order to comprehensively evaluate the quality of salted grass carp, other indicators (K value, EUC, TAV, etc.) should be considered.

### 3.3 Texture properties and color analysis

The texture changes of fish meat are mainly related to the destruction of extracellular matrix structure (collagen) and the resulting changes of intracellular myofibrillar protein (Martinez et al., 2011). Table 2 showed the changes in texture of grass carp meat during brine salting with different salt additions. The initial hardness of grass carp meat was 545.75 g, which increased significantly of salting in 6%, 8%, and 10% salt additions and reached the maximum at 24 hr (747.00 g, 762.42 g, and 796.16 g, respectively). After that, the hardness gradually decreased, which was contrary to the trends of pH in this study. Hultmann and Rustad (2004) reported that the texture properties of fish, such as hardness and springiness, were affected by some factors including myofibrils decomposition and the rate of pH decline. For fish products, texture, especially hardness, was an important quality factor affecting the acceptability of fish. In addition, during the same salting time, the greater the salt addition was, the greater the hardness was. The increase in hardness may be related to and calcium and magnesium ions in salt and the low pH of muscles (Lauritzsen, Akse, Gundersen, & Olsen, 2004). The changes of chewiness of meat were consistent with the hardness. They also reached the peak values at 24 hr of salting with 6%, 8%, and 10% salt additions, which were 248.95, 250.83, and 266.50 g, respectively. In the process of salting, proteins of fish will be denatured due to the action of salt, resulting in the reduction of their own gel properties and the increase of the toughness of tissue structure, which is manifested as the decrease of fish springiness (Hwang et al., 2012). The springiness of grass carp meat increased at the initial stage of salting with 6% salt additions, reached its maximum value of 0.89 at 4 hr, and then gradually decreased. However, during the salting with 8% and 10% salt additions, the springiness was continuously decreased at different rates, indicating that low‐salt treatment could improve the springiness and taste of grass carp meat to some extent. Bahuaud, Gaarder, Veiseth‐Kent, and Thomassen (2010) reported that the decrease in springiness may be due to the action of cathepsin.

**TABLE 2:** Changes in texture properties and color of grass carp meat during brine salting

Salt addition	Salting time (h)	Hardness (g)	Springiness	Cohesiveness	Chewiness (g)	Resilience	L*	a*	b*
6%	0	545.75 ± 35.82^a^	0.88 ± 0.02^cd^	0.42 ± 0.02^bc^	187.11 ± 7.27^a^	0.23 ± 0.01^c^	43.93 ± 1.05^a^	−0.63 ± 0.15^b^	4.77 ± 0.81^a^
	4	575.11 ± 29.62^b^	0.89 ± 0.01^d^	0.50 ± 0.01^e^	192.10 ± 9.00^a^	0.27 ± 0.01^e^	46.90 ± 0.82^b^	−1.27 ± 0.35^a^	3.73 ± 0.90^a^
	8	650.24 ± 17.76^c^	0.89 ± 0.01^d^	0.43 ± 0.01^c^	205.68 ± 1.86^b^	0.27 ± 0.01^e^	43.27 ± 0.29^a^	−1.77 ± 0.40^a^	3.47 ± 0.85^a^
	16	682.09 ± 8.48^de^	0.87 ± 0.01^c^	0.42 ± 0.02^bc^	208.02 ± 8.32^bc^	0.24 ± 0.01^d^	49.50 ± 1.47^c^	−1.87 ± 0.55^a^	5.13 ± 1.10^a^
	24	747.00 ± 33.27^f^	0.84 ± 0.02^b^	0.41 ± 0.01^b^	248.95 ± 6.43^d^	0.18 ± 0.01^b^	46.93 ± 1.10^b^	−1.47 ± 0.15^a^	4.70 ± 0.56^a^
	36	691.21 ± 10.15^e^	0.81 ± 0.01^a^	0.38 ± 0.01^a^	215.33 ± 8.16^c^	0.15 ± 0.01^a^	48.10 ± 1.30^b^	−1.40 ± 0.26^a^	7.20 ± 1.21^b^
	48	660.08 ± 5.29^cd^	0.81 ± 0.01^a^	0.46 ± 0.02^d^	210.68 ± 6.47^bc^	0.15 ± 0.00^a^	46.53 ± 1.45^b^	−0.33 ± 0.21^b^	8.90 ± 1.23^b^
8%	0	545.75 ± 35.82^a^	0.88 ± 0.02^c^	0.42 ± 0.02^a^	187.11 ± 7.27^a^	0.23 ± 0.01^d^	43.93 ± 1.05^a^	−0.63 ± 0.15^b^	4.77 ± 0.81^ab^
	4	660.02 ± 21.27^b^	0.88 ± 0.01^c^	0.50 ± 0.02^c^	200.13 ± 5.58^v^	0.25 ± 0.01^e^	44.43 ± 3.15^a^	−0.57 ± 0.42^b^	5.80 ± 1.73^ab^
	8	673.77 ± 6.12^bc^	0.85 ± 0.02^b^	0.49 ± 0.02^bc^	210.04 ± 2.45^c^	0.24 ± 0.01^e^	42.93 ± 1.21^a^	−0.70 ± 0.17^b^	4.47 ± 0.87^a^
	16	692.07 ± 9.64^cd^	0.84 ± 0.01^ab^	0.48 ± 0.02^bc^	216.65 ± 5.16^cd^	0.21 ± 0.01^c^	47.60 ± 1.21^b^	−0.77 ± 0.12^b^	5.67 ± 0.64^ab^
	24	762.42 ± 7.89^e^	0.81 ± 0.02^a^	0.40 ± 0.02^a^	250.83 ± 2.59^e^	0.16 ± 0.01^ab^	48.40 ± 0.72^b^	−1.67 ± 0.15^a^	6.63 ± 0.49^bc^
	36	699.28 ± 11.40^d^	0.82 ± 0.02^a^	0.47 ± 0.02^b^	220.23 ± 4.45^d^	0.17 ± 0.01^b^	52.03 ± 2.32^c^	−0.07 ± 0.45^c^	8.20 ± 1.73^cd^
	48	663.48 ± 11.86^b^	0.81 ± 0.02^a^	0.48 ± 0.02^bc^	212.01 ± 10.47^c^	0.16 ± 0.01^a^	50.33 ± 1.02^bc^	0.67 ± 0.23^d^	8.77 ± 0.85^d^
10%	0	545.75 ± 35.82^a^	0.88 ± 0.02^b^	0.42 ± 0.02^a^	187.11 ± 7.27^a^	0.23 ± 0.01^e^	43.93 ± 1.05^a^	−0.63 ± 0.15^b^	4.77 ± 0.81^a^
	4	681.40 ± 6.94^b^	0.84 ± 0.04^a^	0.50 ± 0.01^c^	212.17 ± 3.35^d^	0.22 ± 0.01^de^	46.27 ± 1.25^ab^	−1.10 ± 0.03^a^	5.23 ± 1.53^ab^
	8	699.86 ± 9.17^bc^	0.83 ± 0.04^a^	0.54 ± 0.02^d^	221.82 ± 5.41^c^	0.21 ± 0.01^d^	44.00 ± 2.95^a^	−0.83 ± 0.31^ab^	4.67 ± 0.83^a^
	16	701.97 ± 12.29^bc^	0.83 ± 0.01^a^	0.48 ± 0.02^b^	222.54 ± 7.98^c^	0.19 ± 0.01^c^	47.97 ± 0.75^b^	−0.60 ± 0.26^b^	7.07 ± 1.05^bc^
	24	796.15 ± 11.43^e^	0.81 ± 0.03^a^	0.46 ± 0.02^b^	266.50 ± 9.74^d^	0.19 ± 0.01^c^	48.87 ± 1.83^bc^	−0.73 ± 0.25^ab^	6.07 ± 1.67^abc^
	36	724.02 ± 10.21^d^	0.82 ± 0.02^a^	0.46 ± 0.02^b^	217.43 ± 9.11^bc^	0.18 ± 0.01^b^	48.03 ± 0.85^b^	−0.10 ± 0.17^c^	6.60 ± 0.30^abc^
	48	711.98 ± 14.76^cd^	0.82 ± 0.02^a^	0.47 ± 0.02^b^	212.00 ± 7.71^b^	0.16 ± 0.01^a^	50.93 ± 0.68^c^	−0.40 ± 0.20^bc^	7.33 ± 0.75^c^

Note

Different superscript letters in the same column of one salt addition indicate a significant difference (*p* < .05) at different time points.

The results showed that the lightness (L* value) of grass carp meat during brine salting with 6% and 8% salt additions appeared a trend of first increasing and then decreasing, and reached the minimum values of 49.5 at 16 hr and 52.03 at 36 hr, respectively, while the L* value of salted fish in 10% salt additions presented a trend of increasing and reached the maximum value of 50.93 at 48 hr (Table 2). This indicated that salting had an effect on the lightness of grass carp meat, and salting with low salt for a short time was more beneficial to the lightness of grass carp meat. Lauritzsen et al. (2004) reported that the presence of calcium and magnesium ions in salt contributes to the whitening of muscle surfaces. The redness values (a* values) of grass carp salted with three salt additions were negative, and they all decreased first and then increased. However, different salt addition will lead to different time for a* value of grass carp meat to reach the minimum value during salting. They reached the minimum a* values of −1.87 at 16 hr of salting in 6% salt additions, −1.67 at 24 hr of salting in 8% salt additions, and ‐ 1.10 at 4 hr of salting in 10% salt additions, respectively. It can also be seen that the a* value increased with the increasing salt addition, which may be related to the degradation of myoglobin to biliverdin by ring opening at a certain salt concentration in the dark meat of salting fish (Geng et al., 2016). The salting treatment with 6%, 8%, and 10% salt additions increased the yellowness (b* value) of grass carp meat and reached the maximum values at the end of salting (8.90, 8.77, and 7.33, respectively). It can also be found that although the salt treatment increased the b* values, the increase rate of b* values slowed down with the increasing salt addition. A rapid decrease in the pH of fish during salting can also cause color changes (Lauritzsen et al., 2004).

### 3.4 ATP‐related compounds and K value analysis

ATP‐related compounds are important flavor substances in aquatic products, and K value is often used to monitor fish freshness. Adenosine triphosphate (ATP) is mainly found in fresh fish, but ATP will be degraded by endogenous enzymes into adenosine diphosphate (ADP), adenosine phosphate (AMP), and inosine monophosphate (IMP) after fish die. With the extension of death time, IMP degrades into inosine (HxR) and hypoxanthine (Hx) (Howgate, 2005). The changes in the contents of ATP and its related compounds of grass carp meat during brine salting with 6%, 8%, and 10% salt additions for 48 hr were shown in Table 3. ATP‐related compounds are important flavor substances in fish, among which IMP is one of the important factors for the umami taste of fish, while Hx is closely related to the putrefaction of fish (Howgate, 2005). As can be seen from Table 3, ATP contents decreased gradually with the extension of salting time, and the speed of decreasing became slowly with the increase of salt addition during salting. In addition, IMP content of grass carp meat during brine salting was the highest among ATP‐related compounds. Fan et al. (2014) also reached the same conclusion in the determination of ATP and its related compounds in black carp during salting and storage. This was because AMP degraded to HxR very slowly during ATP degradation, which results in the accumulation of IMP in fish (Howgate, 2005). IMP contents of grass carp meat decreased gradually with the extension of salting time during brine salting with 6%, 8%, and 10% salt additions, and decreased significantly at 8, 16, and 8 hr, respectively. After that, the rate of decline in the IMP contents was slowed down as the increase of salt addition and reached the lowest value at the end of salting, 157.62 mg/100 g, 167.85 mg/100 g, and 171.53 mg/100 g, respectively. It was not difficult to see that when grass carp meat were salted in brine with 6%, 8%, and 10% salt additions at 4 hr, the HxR contents significantly decreased to the minimum values of 6.43 mg/100 g, 6.86mg/100g, and 6.92mg/100g, respectively. The change trends in Hx and HxR contents during the salting period of 4–48 hr were opposite to ATP and IMP. Hx is the end product of the ATP degradation pathway. High levels of Hx can cause fish to have a bitter taste of putrefaction, which can make the fish flavor worse (Tersaki, Kajikawa, & Fujita, 1965). Therefore, low‐salt brine salting can delay the putrefying of grass carp meat to some extent.

**TABLE 3:** Changes in the contents and K value of nucleotide compounds and the TAV of IMP and AMP of grass carp meat during brine salting

Salt addition	Salting time (h)	Contents (mg/100g)							K value (%)	TAV	
		IMP	ATP	ADP	AMP	Hx	HxR	Total		IMP	AMP
6%	0	350.08 ± 16.86^d^	3.14 ± 0.76^c^	10.54 ± 1.92^b^	3.03 ± 0.43^c^	3.29 ± 0.52^a^	16.58 ± 1.09^d^	386.66 ± 18.78^e^	5.14 ± 0.10^bc^	14.00 ± 0.67^d^	0.06 ± 0.01^c^
	4	312.84 ± 28.80^d^	2.32 ± 0.44^b^	10.44 ± 1.57^b^	1.95 ± 0.70^ab^	2.65 ± 0.32^a^	6.43 ± 0.25^a^	336.64 ± 30.25^d^	2.71 ± 0.25^a^	12.51 ± 1.15^d^	0.04 ± 0.01^ab^
	8	201.99 ± 34.93^bc^	2.10 ± 0.00^b^	7.10 ± 1.53^a^	1.39 ± 0.76^ab^	1.78 ± 0.34^a^	10.95 ± 2.91^abc^	225.30 ± 33.82^c^	5.86 ± 2.09^bc^	8.08 ± 1.40^bc^	0.03 ± 0.02^ab^
	16	230.76 ± 24.55^c^	2.04 ± 0.39^ab^	8.17 ± 1.53^ab^	2.10 ± 0.25^b^	1.94 ± 0.99^a^	8.33 ± 2.70^ab^	253.33 ± 26.32^bc^	4.05 ± 0.81^ab^	9.23 ± 0.98^c^	0.04 ± 0.01^b^
	24	194.27 ± 18.60^abc^	1.61 ± 0.54^ab^	7.04 ± 1.83^a^	1.90 ± 0.55^ab^	2.18 ± 0.63^a^	15.32 ± 0.75^cd^	222.34 ± 19.37^abc^	7.94 ± 1.12^c^	7.77 ± 0.74^abc^	0.04 ± 0.01^ab^
	36	170.61 ± 6.16^ab^	1.26 ± 0.22^a^	6.70 ± 0.32^a^	1.14 ± 0.16^a^	2.83 ± 1.62^a^	12.62 ± 5.35^bcd^	195.16 ± 7.40^ab^	7.88 ± 3.29^c^	6.82 ± 0.25^ab^	0.02 ± 0.00^a^
	48	157.62 ± 9.82^a^	1.06 ± 0.25^a^	6.70 ± 0.62^a^	1.89 ± 0.21^ab^	5.24 ± 0.21^b^	12.48 ± 0.57^bcd^	185.00 ± 10.62^a^	9.58 ± 0.22^d^	6.30 ± 0.39^a^	0.04 ± 0.00^ab^
8%	0	350.08 ± 16.86^d^	3.14 ± 0.76^ab^	10.54 ± 1.92^d^	3.03 ± 0.43^bc^	3.29 ± 0.52^bc^	16.58 ± 1.09^b^	386.66 ± 18.78^d^	5.14 ± 0.10^bc^	14.00 ± 0.67^d^	0.06 ± 0.01^bc^
	4	308.25 ± 21.97^c^	3.30 ± 1.36^b^	7.25 ± 1.38^ab^	3.57 ± 1.49^c^	2.50 ± 0.97^b^	6.86 ± 0.52^a^	331.73 ± 25.14^c^	2.82 ± 0.16^a^	12.33 ± 0.88^c^	0.07 ± 0.03^c^
	8	272.43 ± 1.63^b^	2.30 ± 0.98^ab^	9.27 ± 0.46^cd^	2.02 ± 0.18^ab^	1.40 ± 0.04^a^	12.01 ± 5.08^ab^	229.44 ± 6.05^b^	4.46 ± 1.62^b^	10.90 ± 0.07^b^	0.04 ± 0.00^ab^
	16	273.56 ± 13.56^b^	2.05 ± 0.51^ab^	9.13 ± 0.68^bcd^	1.93 ± 0.50^ab^	1.47 ± 0.03^a^	12.68 ± 4.58^ab^	300.84 ± 9.84^b^	4.73 ± 1.62^b^	10.94 ± 0.54^b^	0.04 ± 0.01^ab^
	24	191.81 ± 16.63^a^	2.37 ± 0.05^ab^	7.33 ± 0.84^abc^	1.17 ± 0.24^a^	1.62 ± 0.63^ab^	12.95 ± 0.26^ab^	217.25 ± 18.23^a^	6.79 ± 0.25^c^	7.67 ± 0.67^a^	0.02 ± 0.00^a^
	36	184.83 ± 2.73^a^	2.93 ± 0.20^ab^	6.60 ± 0.73^a^	2.22 ± 0.30^ab^	3.78 ± 2.04^c^	11.74 ± 7.08^ab^	212.11 ± 5.42^a^	7.29 ± 2.26^cd^	7.39 ± 0.11^a^	0.04 ± 0.01^ab^
	48	167.85 ± 16.67^a^	1.70 ± 0.76^a^	5.56 ± 0.54^a^	1.35 ± 0.92^a^	3.73 ± 0.92^c^	12.21 ± 0.63^ab^	192.40 ± 19.19^a^	8.28 ± 0.42^d^	6.71 ± 0.67^a^	0.03 ± 0.02^a^
10%	0	350.08 ± 16.86^b^	3.14 ± 0.76^a^	10.54 ± 1.92^b^	3.03 ± 0.43^b^	3.29 ± 0.52^c^	16.58 ± 1.09^b^	386.66 ± 18.78^c^	5.14 ± 0.10^b^	14.00 ± 0.67^b^	0.06 ± 0.01^b^
	4	297.25 ± 34.88^b^	2.96 ± 1.29^a^	8.25 ± 2.38^ab^	2.57 ± 0.99^ab^	2.34 ± 0.72^ab^	6.92 ± 0.51^a^	320.28 ± 34.86^b^	2.91 ± 0.38^a^	11.89 ± 1.40^b^	0.05 ± 0.02^ab^
	8	220.90 ± 67.16^a^	2.54 ± 0.70^a^	5.09 ± 3.11^a^	2.50 ± 1.04^ab^	1.62 ± 0.65^a^	6.95 ± 1.41^a^	239.58 ± 71.35^a^	3.58 ± 0.40^a^	8.84 ± 2.69^a^	0.05 ± 0.02^ab^
	16	229.32 ± 7.02^a^	1.97 ± 0.00^a^	7.15 ± 0.78^a^	1.79 ± 0.45^ab^	1.96 ± 1.15^ab^	9.19 ± 4.65^ab^	251.37 ± 8.83^a^	4.40 ± 2.18^ab^	9.17 ± 0.28^a^	0.04 ± 0.01^ab^
	24	207.32 ± 19.46^a^	2.52 ± 1.15^a^	6.45 ± 0.30^a^	1.66 ± 1.39^ab^	3.44 ± 0.67^c^	9.12 ± 4.83^ab^	230.51 ± 26.23^a^	5.34 ± 1.67^b^	8.29 ± 0.78^a^	0.03 ± 0.03^ab^
	36	173.97 ± 23.47^a^	2.19 ± 0.62^a^	5.81 ± 0.09^a^	1.53 ± 0.65^ab^	2.98 ± 1.53^abc^	12.15 ± 9.19^ab^	198.63 ± 34.97^a^	7.19 ± 3.64^c^	6.96 ± 0.94^a^	0.03 ± 0.01^ab^
	48	171.53 ± 11.50^a^	1.94 ± 0.33^a^	6.24 ± 0.60^a^	1.29 ± 0.31^b^	3.12 ± 0.17^c^	12.24 ± 0.28^a^	196.36 ± 12.06^a^	7.82 ± 0.48^c^	6.86 ± 0.46^a^	0.03 ± 0.01^a^

Note

Different superscript letters in the same column of one salt addition indicate a significant difference (*p* < .05) at different time points.

K value is widely used to evaluate the freshness of fish. The smaller the K value is, the better the freshness is. Generally, K value less than 20% is considered as the first order freshness, indicating that the fish is very fresh (Aubourg, Quitral, & Larrain, 2007). As can be seen from Table 3, K values all showed a trend of first decreasing and then gradually increasing in the salting process. When the grass carp meat were brined with 6%, 8%, and 10% salt additions at 4 hr, K values were significantly reduced to minimum values of 2.71%, 2.82%, and 2.91%, respectively. Since then, the rate of increase in K values became slower with the increase of salt addition. The K values reached the maximum values at 48 hr (9.58%, 8.28%, and 7.82%, respectively), all less than 10%, indicating that the grass carp meat was still very fresh at the end of salting, which was consistent with the results of TVC in this study.

As is known to all, IMP is an important umami nucleotide in fish, which has a synergistic effect with AMP to enhance umami. IMP is also an ideal flavor enhancer with a threshold of 25 mg/100 g, while AMP can inhibit bitter taste and make food produce an ideal sweet and salty taste with a threshold of 50 mg/100 g (Takashi, Maya, & Hideyuki, 2007). TAV is the ratio between the content of flavor substances in the sample and its threshold, and TAV greater than 1 indicates that the substance contributes significantly to the overall taste of the sample. As can be seen from Table 3, the TAVs of IMP in all samples were far greater than those of AMP, and the TAVs of IMP reached minimum values of 6.30, 6.71, and 7.82, respectively at 48 hr, all of which were greater than 1, indicating that IMP contributed significantly to the overall taste of grass carp meat during brine salting.

### 3.5 FAA analysis

Free amino acids are produced by the hydrolysis of protein and are a kind of important flavor substances in aquatic products. The taste characteristics of free amino acids are related to their thresholds and contents. Some free amino acids have obvious taste characteristics when they are high enough in fish meat, such as aspartic acid (Asp), glutamic acid (Glu), glycine (Gly), alanine (Ala), and proline (Pro) are umami and sweet, while lysine (Lys) and histidine (His) are bitter (Shi, Chen, Wan, & Wang, 2014; Wang et al., 2018). Histidine can enhance the flavor of fish and make aquatic products have the characteristics of meat flavor (Deng, Wang, & Liu, 2010). Lioe, Apriyantono, Takara, Wada, and Yasuda (2005) found that some bitter amino acids, such as phenylalanine (Phe) and tyrosine (Tyr), could enhance the umami and sweet of other free amino acids when the content was lower than their taste thresholds. Sulfur taste amino acid itself does not have the meat flavor, but has the important role to the meat flavor formation.

Tables 4, 5, 6 showed the changes in free amino acid contents of grass carp meat during brine salting with 6%, 8%, and 10% salt additions. It can be seen that the contents of Gly, Ala, His, and Pro were relatively high during brine salting, while threonine (Thr), serine (Ser), and leucine (Leu) had little effect on the taste of grass carp meat in the whole salting process due to their contents lower than their thresholds (260 mg/100 g, 150 mg/100 g, and 190 mg/100 g, respectively), which was consistent with the study on changes in free amino acid contents of grass carp in the process of postmortem (Wang et al., 2018). As can be seen from Table 4, in the process of salting with 6% salt additions, the content of Glu increased significantly to the maximum value of 1.60 mg/100 g at 8 hr and then decreased slightly. The contents of Gly, Ala, and His decreased significantly at 0–8 hr, while the maximum value of Pro reached 25.84 mg/100 g at 4 hr. From Table 5, in the process of salting with 8% salt additions, the content of Glu increased significantly to the maximum value of 1.84 mg/100 g in 4 hr and then gradually decreased. The contents of Gly and Ala showed a decreasing trend during the salting process of 8% salt additions, while the contents of His decreased after 8 hr, and the contents of Pro decreased significantly during 0–8 hr. From Table 6, during brine salting with 10% salt additions, the content of Glu increased significantly to the maximum value of 1.54 mg/100 g at 8 hr, while the contents of Gly, Ala, and His decreased in the whole salting process. Therefore, it can be seen that different salt additions had different effects on different free amino acids, and there were differences in the whole salting process of grass carp meat. In addition, by comparing the three tables, it can be found that the total content of umami and sweet amino acids (TUSAA), bitter amino acids (TBAA), and free amino acids (TFAA) decreased significantly in salting of the three salt additions, and the decrease was relatively slow in salting of 8% salt additions. It was further proved that salt additions had effects on the free amino acid contents in grass carp meat, and there were differences in the free amino acid contents during different salting times. Studies have shown that temperature can cause differences in free amino acid contents (Hwang, Chen, Shiau, & Jeng, 2000).

**TABLE 4:** Changes in the contents of free amino acids of grass carp meat during brine salting with 6% salt additions

Amino acid species	Taste characteristics	Threshold (mg/100g)	Contents（mg/100g）						
			0 hr	4 hr	8 hr	16 hr	24 hr	36 hr	48 hr
Asp^★^	fresh/sweet (+)	3	0.58 ± 0.02^c^	0.59 ± 0.06^c^	0.44 ± 0.00^a^	0.48 ± 0.01^ab^	0.45 ± 0.05^ab^	0.52 ± 0.03^bc^	0.51 ± 0.01^abc^
Thr^▲^	bitter (‐)	260	8.25 ± 0.04^c^	5.21 ± 0.19^b^	4.83 ± 0.02^ab^	5.38 ± 0.32^b^	4.79 ± 0.21^ab^	4.77 ± 0.08^ab^	4.44 ± 0.51^a^
Ser ^★^	sweet (+)	150	2.23 ± 0.04^c^	1.74 ± 0.02^b^	1.52 ± 0.20^ab^	1.43 ± 0.07^ab^	1.58 ± 0.23^ab^	1.57 ± 0.04^ab^	1.33 ± 0.15^a^
Glu ^★^	fresh (+)	30	1.23 ± 0.02^ab^	1.46 ± 0.09^bc^	1.60 ± 0.02^c^	1.18 ± 0.07^ab^	1.59 ± 0.27^c^	1.39 ± 0.08^bc^	0.94 ± 0.07^a^
Gly ^★^	sweet (+)	130	69.14 ± 1.26^d^	57.96 ± 1.17^c^	38.39 ± 0.13^ab^	36.00 ± 3.12^ab^	42.34 ± 8.37^b^	34.46 ± 0.13^ab^	32.10 ± 2.95^a^
Ala ^★^	sweet (+)	60	22.47 ± 0.36^c^	18.13 ± 0.86^b^	12.35 ± 0.50^a^	13.77 ± 1.21^a^	14.01 ± 2.21^a^	12.49 ± 0.05^a^	11.33 ± 1.14^a^
Val ^▲^	sweet/bitter (‐)	40	5.83 ± 0.05^cd^	6.68 ± 0.43^d^	4.83 ± 0.65^abc^	4.25 ± 0.15^ab^	5.07 ± 0.62^bc^	5.08 ± 0.24^bc^	3.80 ± 0.52^a^
Met	bitter/ sweet/surfur (‐)	30	0.79 ± 0.07^a^	1.08 ± 0.23^b^	1.42 ± 0.13^c^	1.49 ± 0.02^c^	1.50 ± 0.09^c^	1.59 ± 0.02^c^	1.48 ± 0.06^c^
Ile ^▲^	bitter (‐)	90	4.62 ± 0.08^bc^	5.07 ± 0.53^c^	4.24 ± 0.23^ab^	3.99 ± 0.06^ab^	4.07 ± 0.16^ab^	4.21 ± 0.31^ab^	3.69 ± 0.07^a^
Leu ^▲^	bitter (‐)	190	6.64 ± 0.16^cd^	7.25 ± 0.27^d^	5.72 ± 0.11^ab^	5.71 ± 0.16^ab^	6.28 ± 0.51^bc^	5.86 ± 0.14^ab^	5.58 ± 0.25^a^
Tyr	bitter (‐)	ND	3.55 ± 0.46^a^	5.01 ± 0.99^b^	5.31 ± 0.89^b^	4.70 ± 0.02^ab^	4.27 ± 0.51^ab^	5.53 ± 0.06^b^	5.40 ± 0.03^b^
Phe ^▲^	bitter (‐)	90	1.53 ± 0.57^a^	4.49 ± 0.30^b^	4.35 ± 0.16^b^	4.96 ± 0.49^b^	5.12 ± 0.52^b^	4.98 ± 0.03^b^	4.78 ± 0.04^b^
Lys ^▲^	sweet/ bitter (‐)	50	10.12 ± 0.13^c^	9.29 ± 0.16^c^	6.61 ± 0.20^b^	5.98 ± 0.30^b^	6.21 ± 0.19^b^	5.91 ± 0.18^b^	5.01 ± 0.86^a^
His ^▲^	bitter (‐)	20	192.20 ± 2.65^d^	176.00 ± 9.32^c^	152.75 ± 6.33^b^	163.73 ± 10.70^bc^	150.92 ± 5.45^b^	148.22 ± 1.94^b^	125.19 ± 5.68^a^
Arg ^▲^	sweet/ bitter (‐)	50	1.35 ± 0.02^d^	1.15 ± 0.01^bc^	0.92 ± 0.02^a^	0.98 ± 0.03^ab^	1.01 ± 0.03^abc^	1.17 ± 0.10^c^	0.96 ± 0.16^a^
Pro ^★^	sweet/ bitter (+)	300	25.56 ± 0.02^c^	25.84 ± 0.78^c^	15.08 ± 0.56^b^	14.80 ± 0.59^b^	15.60 ± 2.33^b^	15.91 ± 0.53^b^	10.17 ± 2.40^a^
TUSAA			121.20 ± 1.64^d^	105.72 ± 2.79^c^	69.38 ± 0.15^ab^	67.66 ± 4.92^ab^	75.57 ± 13.46^b^	66.34 ± 0.76^ab^	56.39 ± 6.72^a^
TBAA			230.55 ± 3.69^c^	215.14 ± 9.54^c^	184.26 ± 7.24^b^	194.98 ± 12.20^b^	183.47 ± 6.58^b^	180.19 ± 2.40^b^	153.46 ± 8.08^a^
TFAA			356.09 ± 5.72^c^	326.95 ± 13.55^c^	260.37 ± 8.42^b^	268.82 ± 17.08^b^	264.81 ± 20.64^b^	253.66 ± 3.08^b^	216.73 ± 14.77^a^
TUSAA/ TFAA			34.04 ± 0.09^c^	32.35 ± 0.49^c^	26.66 ± 0.81^ab^	25.16 ± 0.23^a^	28.42 ± 2.87^b^	26.15 ± 0.02^ab^	25.97 ± 1.33^ab^

Note

★indicates fresh, sweet amino acids; ▲indicates bitter amino acids; ND indicates that the threshold was not detected; different lowercase letters “a” and “b” in the same row show that the index was significant different (*p* < .05) at different time points. The same below.

**TABLE 5:** Changes in the contents of free amino acids of grass carp meat during brine salting with 8% salt additions

Amino acid species	Taste characteristics	Threshold (mg/100g)	Contents（mg/100g）						
			0 hr	4 hr	8 hr	16 hr	24 hr	36 hr	48 hr
Asp^★^	fresh/sweet (+)	3	0.58 ± 0.02^c^	0.52 ± 0.01^bc^	0.51 ± 0.02^b^	0.42 ± 0.03^a^	0.53 ± 0.06^bc^	0.46 ± 0.00^ab^	0.52 ± 0.01^bc^
Thr^▲^	bitter (‐)	260	8.25 ± 0.04^c^	6.02 ± 0.08^b^	6.08 ± 0.09^b^	4.83 ± 0.58^a^	4.76 ± 0.35^a^	4.46 ± 0.31^a^	4.88 ± 0.01^a^
Ser ^★^	sweet (+)	150	2.23 ± 0.04^c^	1.81 ± 0.06^b^	1.60 ± 0.19^ab^	1.45 ± 0.09^a^	1.62 ± 0.15^ab^	1.40 ± 0.02^a^	1.39 ± 0.06^a^
Glu ^★^	fresh (+)	30	1.23 ± 0.02^ab^	1.84 ± 0.18^c^	1.40 ± 0.19^b^	1.26 ± 0.29^ab^	1.09 ± 0.01^ab^	0.91 ± 0.02^a^	1.01 ± 0.02^a^
Gly ^★^	sweet (+)	130	69.14 ± 1.26^d^	55.87 ± 4.33^c^	46.25 ± 3.61^b^	38.13 ± 4.76^a^	35.99 ± 4.02^a^	30.70 ± 0.44^a^	33.24 ± 0.43^a^
Ala ^★^	sweet (+)	60	22.47 ± 0.36^e^	19.29 ± 0.02^d^	17.23 ± 1.25^cd^	14.91 ± 1.48^bc^	12.93 ± 1.46^ab^	11.72 ± 0.81^a^	15.03 ± 0.08^bc^
Val ^▲^	sweet/bitter (‐)	40	5.83 ± 0.05^c^	5.82 ± 0.26^c^	5.02 ± 0.14^b^	4.30 ± 0.40^a^	3.88 ± 0.24^a^	3.75 ± 0.28^a^	4.13 ± 0.06^a^
Met	bitter/ sweet/surfur (‐)	30	0.79 ± 0.07^a^	1.60 ± 0.02^c^	1.57 ± 0.02^c^	1.52 ± 0.05^bc^	1.40 ± 0.10^b^	1.47 ± 0.01^bc^	1.58 ± 0.11^c^
Ile ^▲^	bitter (‐)	90	4.62 ± 0.08^a^	4.47 ± 0.24^a^	4.41 ± 0.11^a^	4.70 ± 1.24^a^	4.45 ± 1.20^a^	4.11 ± 0.08^a^	3.75 ± 0.13^a^
Leu ^▲^	bitter (‐)	190	6.64 ± 0.16^d^	6.80 ± 0.02^d^	6.14 ± 0.11^c^	5.79 ± 0.31^bc^	5.44 ± 0.22^ab^	5.33 ± 0.10^a^	5.34 ± 0.08^a^
Tyr	bitter (‐)	ND	3.55 ± 0.46^a^	5.26 ± 0.51^bc^	4.89 ± 0.05^bc^	4.81 ± 0.05^bc^	4.57 ± 0.04^b^	5.27 ± 0.34^bc^	5.43 ± 0.16^c^
Phe ^▲^	bitter (‐)	90	1.53 ± 0.57^a^	4.69 ± 0.31^b^	4.81 ± 0.65^b^	5.41 ± 0.15^b^	5.17 ± 0.00^b^	5.11 ± 0.36^b^	4.92 ± 0.11^b^
Lys ^▲^	sweet/ bitter (‐)	50	10.12 ± 0.13^e^	8.22 ± 0.13^d^	7.05 ± 0.06^c^	6.67 ± 0.51^bc^	5.46 ± 0.49^a^	6.67 ± 0.87^bc^	5.78 ± 0.18^ab^
His ^▲^	bitter (‐)	20	192.20 ± 2.65^d^	166.92 ± 6.60^cd^	180.55 ± 7.74^cd^	137.30 ± 22.69^ab^	154.52 ± 12.84^bc^	125.71 ± 4.14^a^	136.17 ± 1.72^ab^
Arg ^▲^	sweet/ bitter (‐)	50	1.35 ± 0.02^b^	1.11 ± 0.00^a^	1.11 ± 0.06^a^	0.99 ± 0.19^a^	1.04 ± 0.10^a^	0.94 ± 0.10^a^	1.06 ± 0.01^a^
Pro ^★^	sweet/ bitter (+)	300	25.56 ± 0.02^d^	21.67 ± 0.28^c^	16.52 ± 1.55^b^	15.03 ± 1.88^b^	10.55 ± 0.01^a^	10.43 ± 1.01^a^	16.58 ± 0.11^b^
TUSAA			121.20 ± 1.64^e^	101.00 ± 4.32^d^	83.50 ± 6.81^c^	71.19 ± 8.53^b^	62.71 ± 5.24^ab^	55.64 ± 2.26^a^	67.77 ± 0.15^ab^
TBAA			230.55 ± 3.69^c^	204.05 ± 6.28^bc^	215.17 ± 7.94^c^	170.00 ± 26.05^a^	184.72 ± 12.96^ab^	156.09 ± 6.24^a^	166.03 ± 1.78^a^
TFAA			356.09 ± 5.72^c^	311.91 ± 10.07^b^	305.13 ± 1.20^b^	247.51 ± 34.59^a^	253.41 ± 18.07^a^	218.46 ± 8.17^a^	240.81 ± 1.36^a^
TUSAA/ TFAA			34.04 ± 0.09^d^	32.37 ± 0.34^d^	27.37 ± 2.34^bc^	28.80 ± 0.58^c^	24.74 ± 0.30^a^	25.47 ± 0.08^ab^	28.14 ± 0.22^c^

**TABLE 6:** Changes in the contents of free amino acids of grass carp meat during brine salting with 10% salt additions

Amino acid species	Taste characteristics	Threshold (mg/100g)	Contents（mg/100g）						
			0 hr	4 hr	8 hr	16 hr	24 hr	36 hr	48 hr
Asp^★^	fresh/sweet (+)	3	0.58 ± 0.02^c^	0.48 ± 0.08^ab^	0.46 ± 0.01^ab^	0.43 ± 0.04^a^	0.45 ± 0.01^ab^	0.53 ± 0.00^bc^	0.48 ± 0.01^ab^
Thr^▲^	bitter (‐)	260	8.25 ± 0.04^e^	5.75 ± 0.84^d^	5.63 ± 0.07^cd^	4.96 ± 0.00^bcd^	4.00 ± 0.41^ab^	4.69 ± 0.44^abc^	3.81 ± 0.13^a^
Ser ^★^	sweet (+)	150	2.23 ± 0.04^d^	1.77 ± 0.29^c^	1.61 ± 0.07^abc^	1.70 ± 0.07^bc^	1.40 ± 0.13^ab^	1.54 ± 0.09^abc^	1.29 ± 0.02^a^
Glu ^★^	fresh (+)	30	1.23 ± 0.02^b^	1.33 ± 0.07^b^	1.54 ± 0.14^c^	1.20 ± 0.08^b^	0.97 ± 0.14^a^	1.13 ± 0.05^ab^	1.11 ± 0.03^ab^
Gly ^★^	sweet (+)	130	69.14 ± 1.26^d^	54.07 ± 8.57^c^	43.95 ± 4.97^bc^	48.73 ± 0.33^bc^	47.19 ± 5.16^bc^	39.98 ± 4.05^ab^	31.47 ± 0.79^a^
Ala ^★^	sweet (+)	60	22.47 ± 0.36^d^	16.71 ± 2.60^c^	14.86 ± 0.95^bc^	14.92 ± 0.36^bc^	12.57 ± 1.48^ab^	14.23 ± 0.94^bc^	10.29 ± 0.28^a^
Val ^▲^	sweet/bitter (‐)	40	5.83 ± 0.05^c^	4.44 ± 0.49^b^	4.35 ± 0.18^b^	5.62 ± 0.07^c^	4.02 ± 0.52^ab^	3.98 ± 0.28^ab^	3.58 ± 0.10^a^
Met	bitter/ sweet/surfur (‐)	30	0.79 ± 0.07^a^	1.53 ± 0.04^bc^	1.57 ± 0.01^bc^	1.56 ± 0.03^bc^	1.65 ± 0.08^c^	1.50 ± 0.02^b^	1.53 ± 0.01^b^
Ile ^▲^	bitter (‐)	90	4.62 ± 0.08^ab^	5.18 ± 1.28^b^	4.29 ± 0.23^ab^	4.21 ± 0.24^ab^	3.89 ± 0.47^ab^	4.14 ± 0.05^ab^	3.51 ± 0.17^a^
Leu ^▲^	bitter (‐)	190	6.64 ± 0.16^c^	6.62 ± 0.64^c^	6.08 ± 0.23^bc^	5.94 ± 0.01^bc^	5.44 ± 0.42^ab^	5.84 ± 0.22^bc^	4.98 ± 0.09^a^
Tyr	bitter (‐)	ND	3.55 ± 0.46^a^	4.80 ± 0.06^ab^	5.30 ± 0.48^b^	5.26 ± 0.90^b^	4.98 ± 0.54^b^	5.30 ± 0.50^b^	4.75 ± 0.03^ab^
Phe ^▲^	bitter (‐)	90	1.53 ± 0.57^a^	4.65 ± 0.16^b^	5.20 ± 0.54^bc^	4.40 ± 0.14^b^	5.00 ± 0.31^bc^	5.24 ± 0.31^bc^	5.64 ± 0.06^c^
Lys ^▲^	sweet/ bitter (‐)	50	10.12 ± 0.13^d^	4.68 ± 0.37^a^	5.51 ± 0.03^abc^	6.22 ± 0.16^c^	5.06 ± 0.67^ab^	5.81 ± 0.47^bc^	4.65 ± 0.51^a^
His ^▲^	bitter (‐)	20	192.20 ± 2.65^e^	184.26 ± 26.43^de^	168.05 ± 4.18^cde^	155.13 ± 0.79^bcd^	141.84 ± 14.58^abc^	133.63 ± 14.58^ab^	117.45 ± 1.41^a^
Arg ^▲^	sweet/ bitter (‐)	50	1.35 ± 0.02^b^	0.87 ± 0.12^a^	1.00 ± 0.04^a^	0.89 ± 0.02^a^	0.96 ± 0.18^a^	1.04 ± 0.13^a^	1.05 ± 0.01^a^
Pro ^★^	sweet/ bitter (+)	300	25.56 ± 0.02^d^	10.58 ± 1.15^ab^	14.78 ± 1.50^c^	15.90 ± 0.58^c^	12.07 ± 1.84^b^	16.60 ± 1.19^c^	8.81 ± 0.59^a^
TUSAA			121.20 ± 1.64^c^	84.93 ± 12.77^b^	77.21 ± 7.62^b^	82.88 ± 1.46^b^	74.64 ± 8.76^b^	74.00 ± 6.32^b^	53.45 ± 0.09^a^
TBAA			230.55 ± 3.69^d^	216.44 ± 30.34^cd^	200.10 ± 3.81^bcd^	187.36 ± 0.80^bc^	170.20 ± 16.95^ab^	164.38 ± 15.86^ab^	144.68 ± 0.56^a^
TFAA			356.09 ± 5.72^d^	307.69 ± 43.20^cd^	284.18 ± 11.92^bc^	277.06 ± 1.59^bc^	251.47 ± 26.33^ab^	245.18 ± 22.67^ab^	204.40 ± 0.49^a^
TUSAA/ TFAA			34.04 ± 0.09^c^	27.58 ± 0.28^a^	27.14 ± 1.55^a^	29.91 ± 0.36^b^	29.66 ± 0.38^b^	30.19 ± 0.21^b^	26.15 ± 0.11^a^

### 3.6 EUC and TAV analysis

**FIGURE 3:**
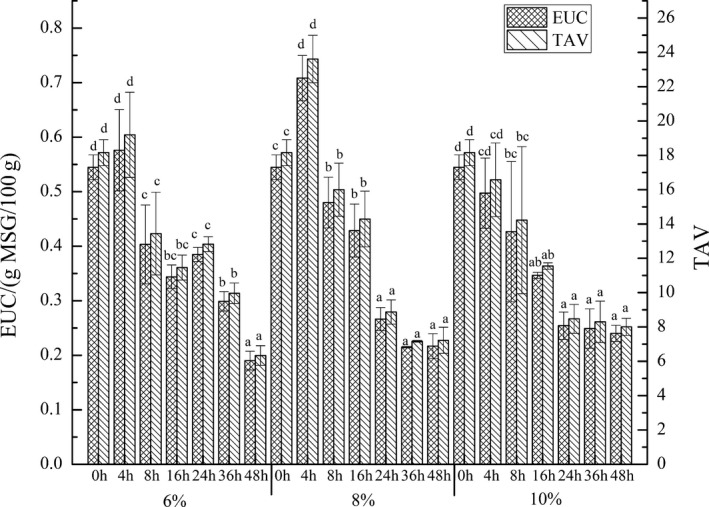
The EUC value and TAV of grass carp meat during brine salting. Data (mean ± *SD*) with different letters in the same salt addition are significantly different (*p* < .05)

EUC is used to reflect the synergistic effect of umami amino acids and flavor nucleotides, indicating the intensity of umami. The main umami amino acids in grass carp are Asp and Glu, which can synergize with IMP and AMP to improve the overall umami of grass carp meat (Shi et al., 2017). Figure 3 showed the changes of EUC and its TAV of grass carp meat during brine salting with 6%, 8%, and 10% salt additions. The taste threshold of MSG is 0.03 g/100 ml. It was not difficult to see that TAVs of EUC in grass carp meat were greater than 1 in salting of three salt additions, indicating that the umami produced by the synergistic action of flavor nucleotides and amino acids contributed significantly to the overall taste of salted grass carp meat. In addition, EUC values showed a trend of first increasing and then decreasing during brine salting with 6% and 8% salt additions, and reached maximum values (0.58 gMSG/100g and 0.71 gMSG/100g, respectively) at 4 hr and minimum values (0.19 gMSG/100 g and 0.22 gMSG/100 g, respectively) at 48 hr. However, EUC value showed a significant decrease during 0–16 hr of brine salting with 10% salt additions, after which the change was not significant. According to the comparison of the EUC values and TAV in all brine salting processes, EUC values and TAV were larger during brine salting for 0–16 hr with 8% salt additions, and the umami was strongest at 4 hr. Therefore, the synergistic effects of flavor nucleotides and amino acids in grass carp meat were different in salting with different salt additions, and the salting time had a great influence on it.

### 3.7 Electronic tongue analysis

**FIGURE 4:**
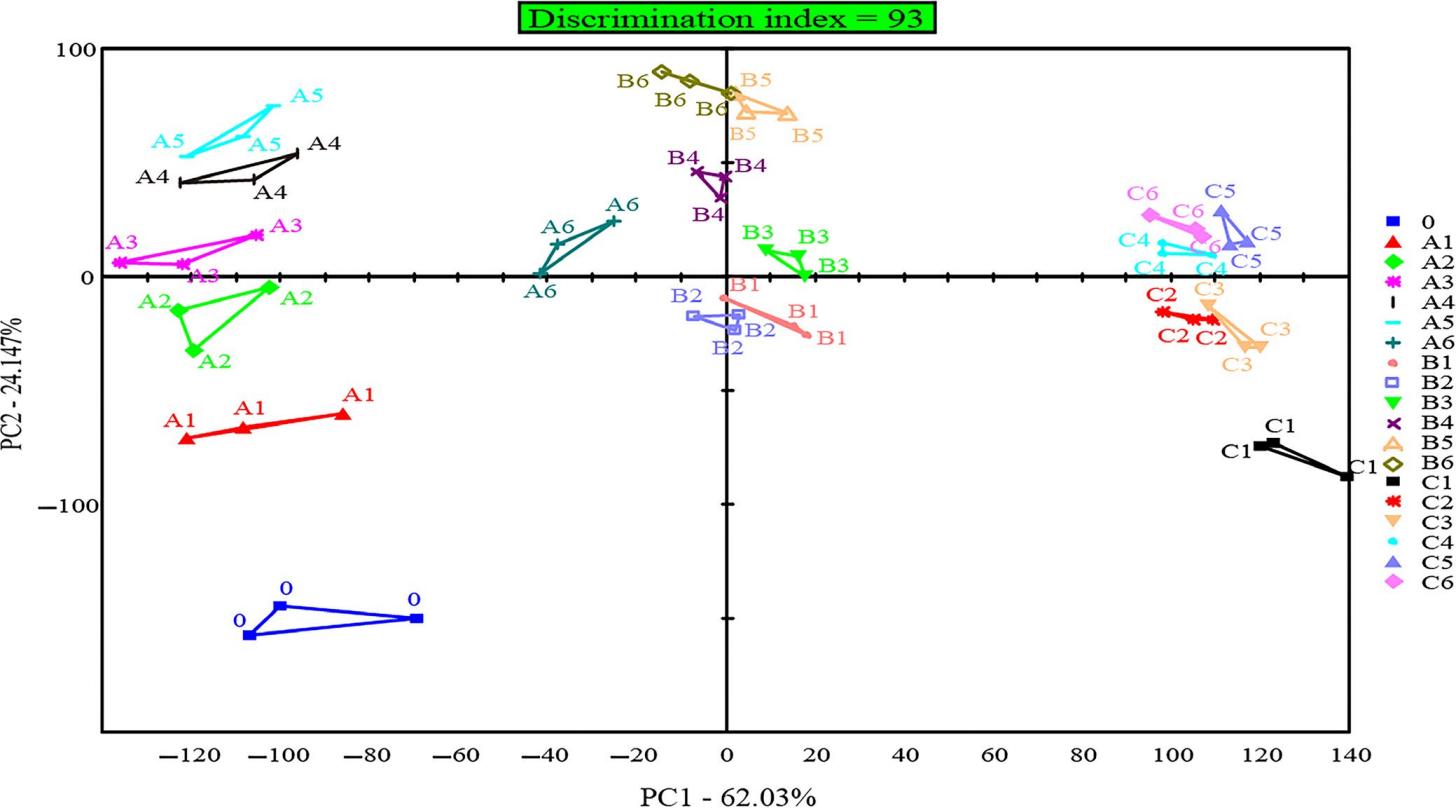
PCA of electronic tongue results recorded of grass carp meat during brine salting. *Note*: 0 represented 0 hr of brine salting, and A1‐A6, B1‐B6, and C1‐C6 represented 4, 8, 16, 24, 36, and 48 hr of brine salting, respectively, for 6%, 8%, and 10% salt additions

The electronic tongue uses a sensor array technology that allows both qualitative and quantitative taste recognition (Beullens et al., 2006). Figure 4 was the principal component analysis (PCA) of the taste of grass carp meat during brine salting by the electronic tongue technology, and the result was a two‐dimensional scatter diagram composed of PC1 and PC2 axes. The contribution rate of principal components in PCA diagram represents the original information contained in it. The larger the contribution rate of cumulative variance (>85%), the more fully it reflects the overall information of samples (Raithore et al., 2015). As can be seen from Figure 4, the contribution rates of the first principal component (PC1) and the second principal component (PC2) were 62.03% and 24.15%, respectively, and the cumulative variance contribution rates were 86.18%, indicating that the PCA diagram based on the electronic tongue could accurately reflect the taste changes of salted grass carp meat. The smaller the distance between the points in the PCA diagram, the smaller the difference. The smaller area of three repeated samples reflects the more stable the recognition of the sample by the electronic tongue (Legin, Rudnitskaya, Vlasov, Di Natale, & D'Amico, 1999). There is no overlapping area in PCA, indicating that the electronic tongue can clearly identify the differences between different samples (Beullens et al., 2006). As can be seen from Figure 4, the principal component identification value (DI) of grass carp meat was 93, and there was no overlap area in the PCA diagram, indicating that the taste of grass carp meat was significantly different in different salting times and salting processes. Further analysis showed that there was a significant difference in the taste profile distribution of grass carp meat with different salt additions, which further indicated that the tastes of grass carp meat salted with 6%, 8%, and 10% salt additions were different.

## 4 CONCLUSIONS

The purpose of this study was to research the quality changes of grass carp meat during brine salting with different salt additions. NaCl contents were proportional to salting time and salt addition, while brine salting had little effect on moisture content of grass carp meat. TVC of grass carp meat increased with the extension of salting time, and the higher salt addition could inhibit the growth of bacteria to some extent. The change trends of hardness of grass carp meat were opposite to pH within 24 hr of salting, and higher salt addition had adverse effects on the hardness and chewiness. Short salting period of low‐salt additions was more beneficial to enhance lightness. In the three salting processes, the grass carp meat was very fresh (K values < 10%) and K values were significantly reduced to the minimum values at 4 hr. Among the three salting processes, the contents of TUSAA, TBAA, and TFAA decreased the most slowly with 8% salt additions. PCA showed that the tastes of grass carp meat were significantly different in all salting samples. Sensory assessment showed that the qualities of grass carp meat salted with 6% and 8% salt additions were better than 10%, and the quality of grass carp meat salted within 16 hr was better. Combined EUC and TAV analysis, grass carp meat salted with 8% salt additions for 4–8 hr had a better taste and should not exceed 16 hr. Therefore, this study suggested that grass carp meat should be brine salting with 8% salt additions and eaten at 4–8 hr. At this point, it can also be processed for the next step, such as steaming or frying, in which the grass carp meat had a better taste and quality. These results would provide useful information for quality control of grass carp during salting and theoretical reference for improving the nutritional value of grass carp. In addition, further exploration of volatile components and endogenous protease of grass carp during brine salting will contribute to the improvement of flavor change mechanism and the optimization of salting process of grass carp.

## CONFLICT OF INTEREST

We declare that we do not have any commercial or associative interest that represents a conflict of interest in connection with the work submitted.
